# MST-Net: a multi-scale spatial transformation network for automated T2/T3 staging of rectal cancer on preoperative T2-weighted MRI

**DOI:** 10.1186/s13244-026-02377-3

**Published:** 2026-09-04

**Authors:** Wei Liu, Ling Yin, Xiong Ran, Tian-Xiang Xu, Yun Du, Jiang-Yi He, Shao-Quan Zhou, Jian-Jun Li

**Affiliations:** 1 Department of Oncology, First Affiliated Hospital of Army Medical University, Chongqing, China; 2 Radiology Department, Chongqing General Hospital, Chongqing University, Chongqing, China; 3 School of Computer Science and Technology, Xi’an Jiaotong University, Xi’an, China

**Keywords:** Rectal neoplasms, Deep learning, Magnetic resonance imaging, Neoplasm staging

## Abstract

**Objective:**

This study aimed to develop a deep learning model (MST-Net) for the segmentation-free automated and precise differentiation between T2 and T3 staging of rectal cancer based on preoperative T2-weighted magnetic resonance imaging (T2WI).

**Materials and methods:**

Preoperative T2WI images and postoperative pathologically confirmed T2/T3 staging labels from rectal cancer patients were retrospectively collected from two centers between January 2020 and January 2025. The core of MST-Net includes: a pyramid cross-stage feature fusion module to integrate shallow texture details and deep semantic information, mitigating the loss of spatial details in deep networks; and a lightweight spatial attention mechanism to adaptively focus on key radiomics features within the tumor region. Model performance was evaluated using the area under the receiver operating characteristic curve (AUC) and accuracy (ACC).

**Results:**

A total of 309 patients were included in the analysis, comprising a training set (*n* = 216) and an internal test set (*n* = 54) from Center 1, and an external independent validation set (*n* = 39) from Center 2. MST-Net achieved an AUC of 0.95 (ACC = 90%) on the internal test set, outperforming junior radiologists (ACC = 69%) and performing comparably to senior radiologists (ACC = 85%). On the external validation set, it achieved an AUC of 0.85 (ACC = 79%), also surpassing junior radiologists (ACC = 65%) and reaching the level of senior radiologists (ACC = 72%).

**Conclusion:**

MST-Net enables segmentation-free automated and high-accuracy prediction of T2/T3 staging in rectal cancer based on routine T2WI images. This model provides a reproducible auxiliary tool for clinical personalized treatment decision-making, potentially reducing the dependence of staging results on physician experience.

**Key Points:**

- **Question** Accurate differentiation between T2 and T3 rectal cancer on preoperative T2-weighted MRI remains clinically challenging but is essential for treatment planning.
- **Findings** MST-Net achieved high diagnostic accuracy and robust discrimination for T2 versus T3 staging in both internal and external validation cohorts.
- **Critical Relevance** This study demonstrates that a segmentation-free deep learning model using routine T2-weighted MRI can enable accurate preoperative T-staging of rectal cancer, supporting standardized and efficient radiological assessment and facilitating more consistent clinical decision-making in rectal cancer management.

**Graphical Abstract:**

**Figure:**
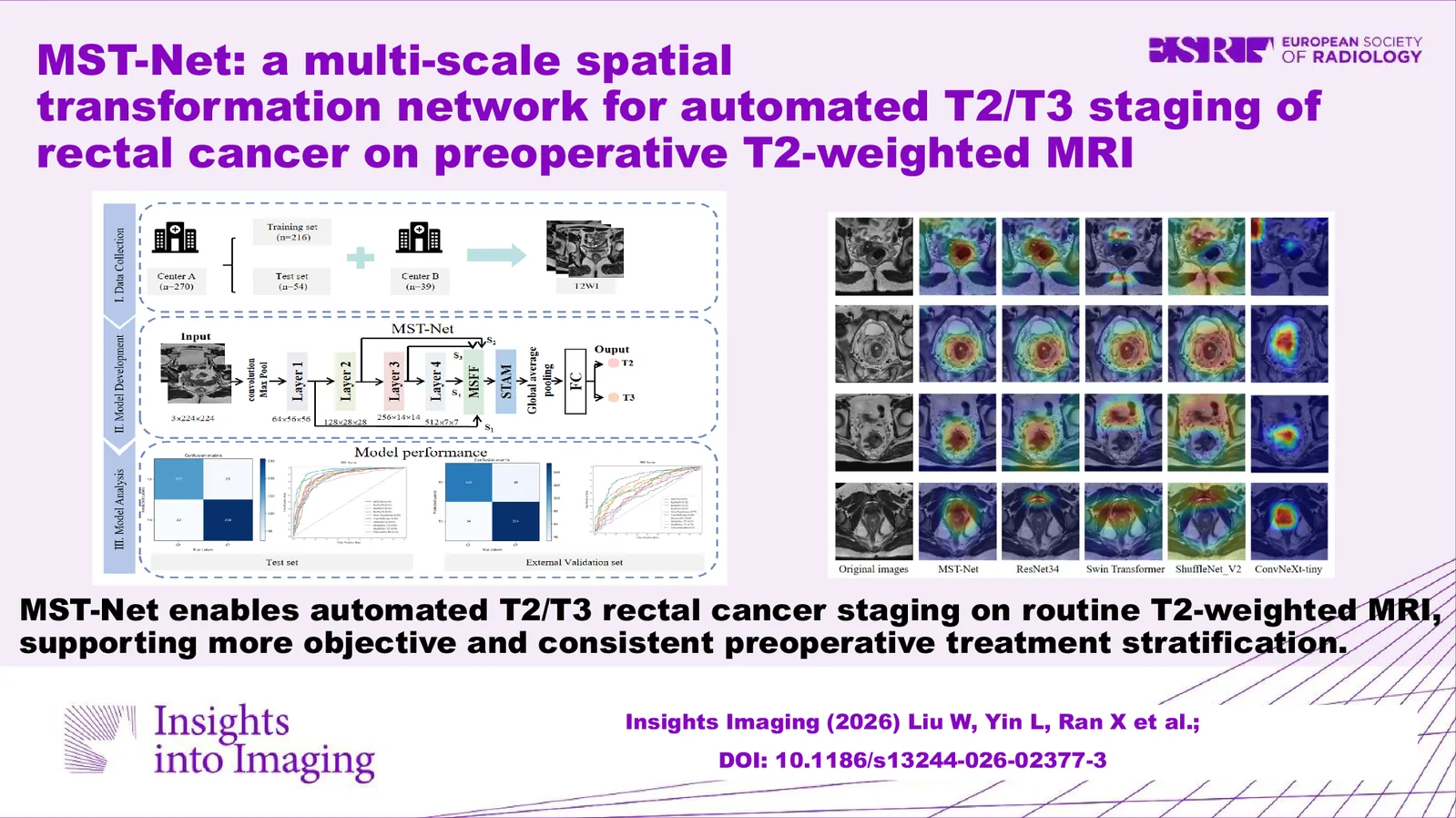

## Introduction

Colorectal cancer (CRC) is a highly prevalent and aggressive malignant tumor of the digestive system worldwide. Global statistics from 2022 reported approximately 1.93 million new CRC cases, which accounted for 9.6% of all newly diagnosed cancers and ranked third in global incidence. During the same period, there were about 904,000 deaths, representing 9.3% of cancer-related mortality, making CRC the second leading cause of cancer death globally [1]. In China, the rise in CRC incidence and mortality is mostly attributed to changes in dietary patterns and lifestyle [2, 3].

For patients diagnosed with rectal cancer, precise preoperative staging is a cornerstone of therapeutic planning, critically influencing decisions regarding neoadjuvant therapy, surgical resection margins, and ultimately, prognostic outcomes [4]. The T-stage component, which evaluates the depth of tumor invasion through the rectal wall and into adjacent structures, is particularly pivotal for stratifying treatment pathways. Clinically, a T2-stage tumor often permits direct radical resection, whereas a T3-stage tumor typically necessitates neoadjuvant chemoradiotherapy to induce tumor downstaging, thereby mitigating local recurrence risks and enhancing surgical success [5, 6]. Consequently, establishing a robust and accurate preoperative distinction, especially at the critical T2/T3 juncture, is of paramount clinical significance.

Magnetic resonance imaging (MRI) offers advantages in evaluating tumor invasion depth, regional lymph node status, and the risk of circumferential resection margin (CRM) involvement, which has established it as one of the most important and reliable imaging modalities for preoperative T-staging of rectal cancer [7]. However, conventional MRI staging heavily relies on the subjective interpretation of morphological signs by radiologists, and its accuracy remains limited with considerable inter-observer variability [8–10]. Variations in radiologists’ expertise can lead to significant discrepancies in staging results [11–13]. Additionally, the manual interpretation process is time-consuming, and its objectivity and reproducibility are constrained. Consequently, there is a pressing need for a robust automated method to enhance the precision, consistency, and objectivity of rectal cancer T-staging.

In recent years, the application of artificial intelligence (AI) in medical image analysis has rapidly expanded, offering new technological pathways for preoperative T-staging of rectal cancer [14, 15]. Previous studies have explored various strategies, including integrating multimodal information and radiomics fusion modeling [16–18], traditional machine learning frameworks incorporating feature selection [19, 20], as well as end-to-end deep learning models and their ensemble approaches [21, 22]. Although these methods have made progress in automating T2/T3 discrimination, common bottlenecks persist: radiomics and machine learning approaches often depend on manual segmentation and handcrafted feature engineering, which are time-consuming and highly sensitive to operator expertise, while their cross-center reproducibility and generalizability can be easily affected by variations in scanning protocols. Conversely, some deep learning methods still face challenges such as limited training data size, insufficient capture of tumor boundaries and local invasion signs by models, and the need for further validation of clinical interpretability and robustness. Based on these limitations, establishing a more robust and generalizable automated imaging model for accurately distinguishing between T2 and T3 stages remains a pressing clinical need.

This paper introduces MST-Net, a multi-scale feature fusion network engineered for the segmentation-free automated and precise T2/T3 staging of rectal cancer. The architecture effectively preserves fine-grained details from shallow layers while integrating rich semantic information from deeper features. Furthermore, the incorporation of a Spatial Transformation Attention Mechanism (STAM) substantially improves the model’s resilience to spatial deformations present in input MRI scans or intermediate feature maps, enabling adaptive focus on tumor-specific regions. Crucially, we hypothesize that by synergistically leveraging these architectural advantages, MST-Net can achieve diagnostic performance comparable to or exceeding that of expert radiologists in distinguishing T2 from T3 stages. Collectively, this methodology facilitates high-accuracy, automated staging, thereby offering a valuable tool for enhancing clinical decision-making processes.

## Materials and methods

### Study population

**Fig. 1:**
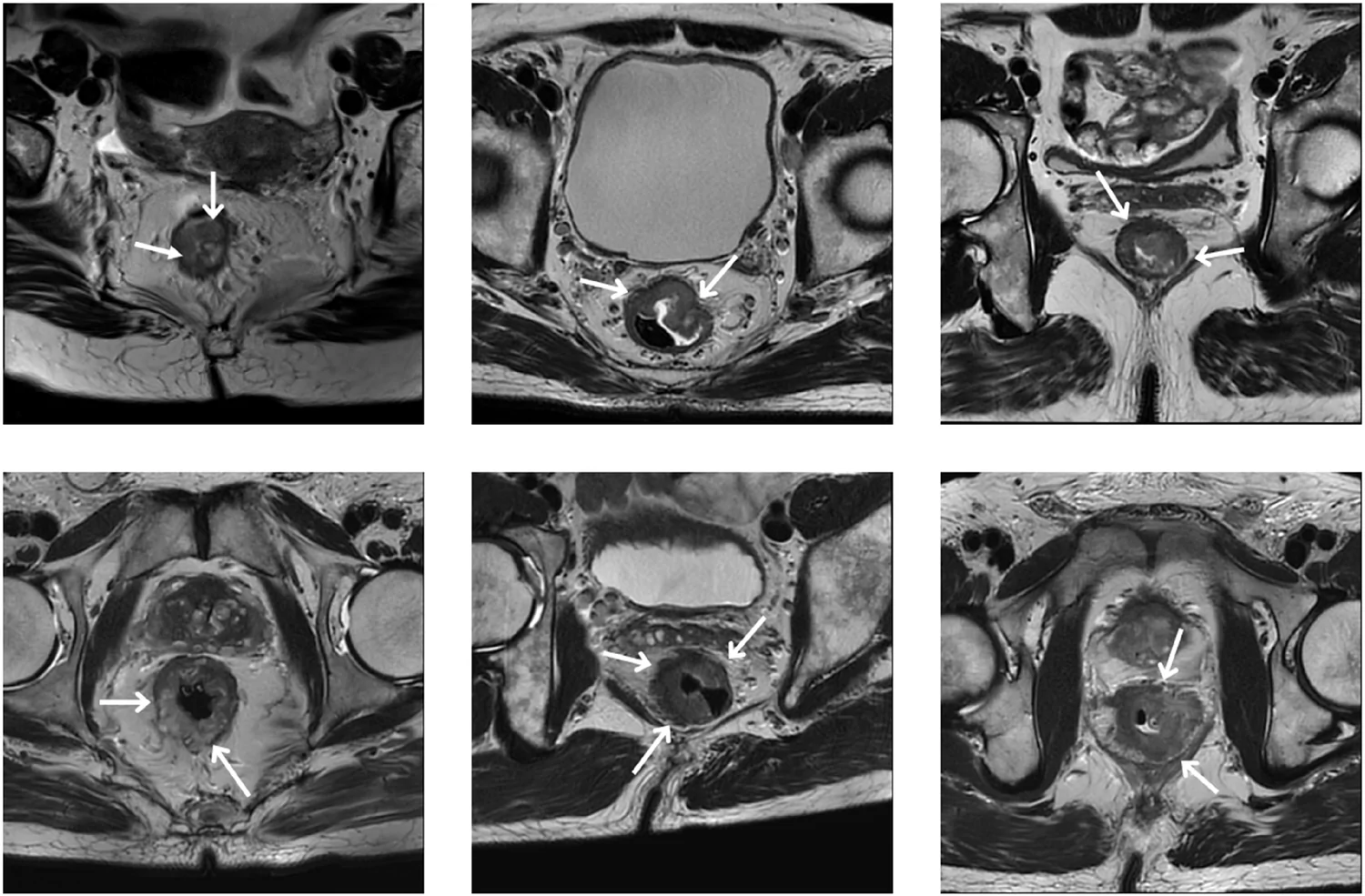
Representative T2-weighted imaging (T2WI) examples of rectal cancer. The first and second rows show tumors pathologically confirmed as T2 and T3 stages, respectively

**Fig. 2:**
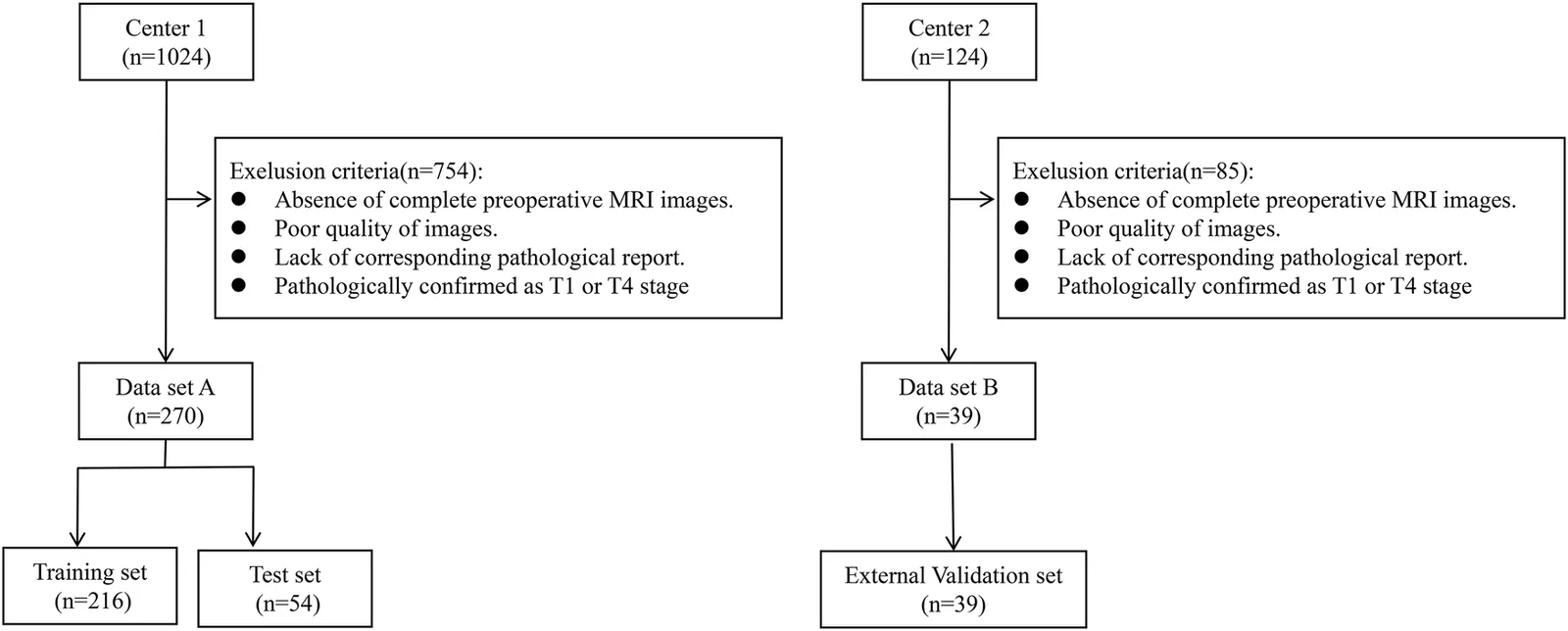
Data collection flowchart

This study used multicenter datasets to develop and validate the proposed model. Dataset A, sourced from Center 1, was utilized for model development, encompassing both training and internal testing phases. In contrast, Dataset B, obtained from Center 2, was reserved exclusively for independent external validation to rigorously assess generalizability. The specific composition was as follows: Dataset A consisted of a retrospectively curated cohort of 270 patients with rectal cancer from Center 1 (June 2022 to January 2025), providing 2486 magnetic resonance images. This included 110 T2-stage cases (1080 images) and 160 T3-stage cases (1406 images). Dataset B comprised a retrospective analysis of 39 patients from Center 2 (January 2020 to January 2025), yielding 346 images, with 16 T2-stage (154 images) and 23 T3-stage (192 images) cases. The T-stage for all enrolled cases was pathologically confirmed, serving as the diagnostic gold standard. Furthermore, all included MRI sequences were required to meet stringent quality criteria, ensuring clarity and the absence of significant motion artifacts or metallic distortions. Each image also had to distinctly show a rectal tumor. Representative T2WI examples for both T2 and T3 stages are illustrated in Fig. 1. The inclusion criteria were as follows: (1) patients who underwent surgical resection for rectal cancer with postoperative pathological confirmation of T2 or T3 stage; (2) no history of preoperative pelvic radiotherapy, chemotherapy, or targeted therapy; and (3) availability of complete preoperative imaging and clinical data. The exclusion criteria included: (1) incomplete preoperative MRI sequences; (2) images of insufficient quality to enable analysis; and (3) lack of a corresponding pathology report for verification. The data collection process is summarized in Fig. 2. From Dataset A (Center 1), patients were randomly allocated to a training set and a testing set in an 8:2 ratio, with further details provided in the Supplementary Material (Table S1). This study was conducted in accordance with the principles of the Declaration of Helsinki and was approved by the Ethics Committee of the First Affiliated Hospital of Army Medical University, PLA (Approval Number: (B) KY2025272).

### MRI scanning methods

Routine T2WI examinations were performed in both centers. Center 1 used a United Imaging uMR770 3.0 T (2022) MRI scanner, while Center 2 employed a SIEMENS Magnetom Skyra 3.0 T (2020) MRI scanner; both systems were equipped with abdominal phased-array coils. Before the examination, patients underwent bowel preparation, including overnight fasting. All patients were positioned supine during the scans. Appropriate abdominal compression was applied to minimize motion artifacts. The specific scanning parameters for each center are outlined in Table 1. All magnetic resonance imaging (MRI) images were exported in JPG format, and slices containing tumors were selected by a radiologist with over 15 years of clinical experience.

**Table 1:** Key parameters for rectal MRI scanning

MRI machine model	United Imaging uMR770	SIEMENS Magnetom Skyra
Magnetic field strength	3.0 T	3.0 T
Echo train length	23	16
Field of view (mm²)	180 × 180	180 × 180
Slice thickness (mm)	3.0	3.0
Matrix size	256 × 256	320 × 320
Repetition time/echo time (ms)	5097/109.2	4000/108
Bandwidth (Hz/pixel)	270	110
Flip angle (°)	130	160
Acquisition time	3 min 04 s	4 min 10 s

### Construction of the MST-Net model

**Fig. 3:**
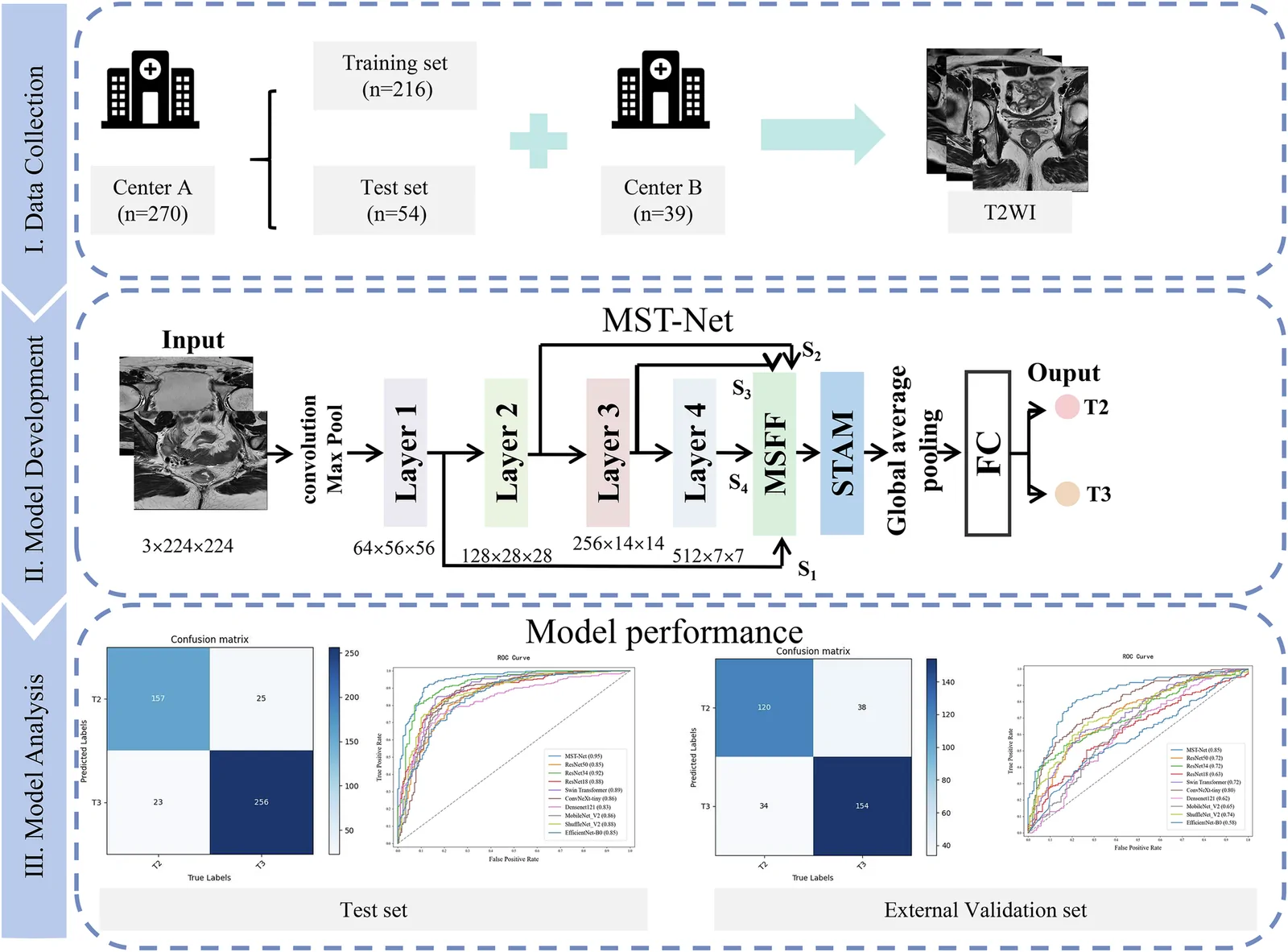
Overall workflow diagram of this study. MSFF, multi-scale feature fusion; STAM, spatial transformation attention mechanism; FC, fully connected layer

This study presents a deep convolutional neural network named MST-Net, which is grounded in a multi-scale spatial transformation architecture. The model is designed to enhance image classification performance through two core components: multi-scale feature fusion (MSFF) and a spatial transformation attention mechanism (STAM). As illustrated in Fig. 3, the overall architecture encompasses input image processing, feature extraction, multi-scale fusion, attention enhancement, and classification output. Before being fed into the network, all input images were resized to 224 × 224 pixels to ensure a uniform input dimension. During model training, data augmentation was performed to improve model robustness. The augmentation strategies included random flipping and contrast adjustment. MST-Net was designed as a segmentation-free automated classification model after image input. Unlike radiomics-based or ROI-based methods, it does not require manual tumor segmentation, ROI delineation, or handcrafted feature extraction. Tumor-containing pelvic T2WI slices are directly uploaded to the network, and MST-Net automatically extracts multi-scale features to generate the final T2/T3 staging prediction results.

A pivotal innovation of our model is the pyramid-based, cross-stage feature fusion module. This module begins by applying adaptive average pooling to normalize feature maps from different hierarchical layers to a unified 7 × 7 spatial resolution. Subsequently, 1 × 1 convolutional layers adjust the channel dimensionality at each stage, reducing them to one-quarter of the total output channels. The refined feature maps are then concatenated along the channel dimension, and a 3 × 3 convolution is employed to foster cross-scale feature interaction. This design effectively alleviates the typical limitation in deep networks where enhanced semantic information in deeper layers comes at the cost of diminished spatial detail from shallower representations. Detailed MSFF methods are provided in the Supplementary Material (Appendix E1 and Appendix Fig. S1).

Additionally, a lightweight attention module inspired by the Spatial Transformer is integrated. It adopts a channel compression strategy (512 → 128 → 1) to produce a spatial weight map, which is normalized via a Sigmoid function. The normalized weights are applied through element-wise multiplication to the fused features, dynamically accentuating responses in anatomically critical regions of the input image. Detailed STAM methods are provided in the Supplementary Material (Appendix E2 and Fig. S2).

Collectively, the MSFF mechanism ensures the integration of detailed, shallow-edge features with high-level, deep semantic features. Complementing this, the STAM enhances the model’s focus on diagnostically relevant regions. This combined approach improves the sensitivity to subtle distinctions between T2 and T3 stage rectal cancer in T2WI.

### Experimental environment and parameter settings

The experimental hardware configuration used in this research includes an Intel® Core™ i5-14450HX CPU, an NVIDIA GeForce RTX 4060 GPU with 8.0 GB of dedicated GPU memory. The software configuration is as follows: the operating system is Windows 11, the development environment is PyCharm, the runtime environment is Python 3.9, CUDA version 11.3, and the deep learning framework is PyTorch 1.12.1. During model training, the Area under the receiver operating characteristic curve (AUC) on the validation set was monitored after each epoch. A model selection strategy based on the maximum validation AUC was adopted. The model parameters corresponding to the epoch with the highest validation AUC were retained as the final model for subsequent evaluation. Detailed parameters of MST-Net are provided in the Supplementary Material (Table S2).

### Statistical analysis

All statistical analyses were conducted using Python (version 3.9), with primary dependencies on the scikit-learn, statsmodels, scipy, pandas, numpy, and matplotlib libraries. Quantitative data with a normal distribution are presented as the mean ± standard deviation (x̄ ± s), while categorical variables are expressed as frequencies and percentages (%). To objectively assess the model’s classification performance, we employed the following evaluation metrics: accuracy (Acc), precision (Pre), recall (Rec), specificity (Spe), F1 score, area under the receiver operating characteristic curve (AUC), and the receiver operating characteristic (ROC) curve. The discriminative ability of the MST-Net model was compared with control models using the DeLong test, and the McNemar test was applied to evaluate diagnostic discrepancies between MST-Net and assessments by three physicians. *p* < 0.05 was considered statistically significant. Definitions for these metrics can be found in the Supplementary Material (Appendix E3).

## Results

### Baseline characteristics of the study cohorts

A total of 270 patients with rectal cancer were included in Dataset A, and 39 patients were included in Dataset B. In Dataset A, the mean age was 60.7 ± 11.4 years, and 171 patients were male (63.3%). Pathologic T2 and T3 stages accounted for 110 cases (40.7%) and 160 cases (59.3%), respectively. The distributions of pathologic N stage were N0 in 147 patients (54.4%), N1 in 67 patients (24.8%), and N2 in 56 patients (20.7%). Tumors were located in the low, middle, and high rectum in 108 patients (40.0%), 125 patients (46.3%), and 37 patients (13.7%), respectively. Serum CEA levels were ≤ 5 ng/mL in 162 patients (60.0%) and > 5 ng/mL in 108 patients (40.0%). In Dataset B, the mean age was 64.2 ± 13.6 years, and 23 patients were male (59.0%). The numbers of pathologic T2 and T3 cases were 16 (41.0%) and 23 (59.0%), respectively. Pathologic N0, N1, and N2 stages were found in 21 patients (53.8%), 10 patients (25.6%), and 8 patients (20.5%), respectively. Low-, middle-, and high-rectal tumors were observed in 7 patients (17.9%), 21 patients (53.8%), and 11 patients (28.2%), respectively. Serum CEA levels were ≤ 5 ng/mL in 25 patients (64.1%) and > 5 ng/mL in 14 patients (35.9%). Detailed demographic and clinicopathologic characteristics are summarized in Table 2.

**Table 2:** Patient characteristics of Dataset A and Dataset B

	Dataset A	Dataset B
Characteristic	Patients (*n* = 270)	Patients (*n* = 39)
Mean age (years)*	60.7 ± 11.4	64.2 ± 13.6
Sex		
Male	171 (63.3)	23 (59.0)
Female	99 (36.7)	16 (41.0)
Pathologic T stage		
T2	110 (40.7)	16 (41.0)
T3	160 (59.3)	23 (59.0)
Pathologic N stage		
N0	147 (54.4)	21 (53.8)
N1	67 (24.8)	10 (25.6)
N2	56 (20.7)	8 (20.5)
Location of rectal cancer		
Low	108 (40.0)	7 (17.9)
Middle	125 (46.3)	21 (53.8)
High	37 (13.7)	11 (28.2)
CEA		
≤ 5 ng/mL	162 (60.0)	25 (64.1)
> 5 ng/mL	108 (40.0)	14 (35.9)

Unless otherwise indicated, data are numbers of patients, with percentages in parentheses

*CEA* carcinoembryonic antigen

* Data are means ± SDs

### Model results

The performance of MST-Net was further evaluated using five-fold cross-validation based on Dataset A, with the results summarized in the Supplementary Material (Table S3). MST-Net achieved a mean accuracy of 86.8% ± 2.0%, mean precision of 90.0% ± 1.3%, mean recall of 88.2% ± 2.1%, mean specificity of 84.6% ± 1.6%, and mean F1 score of 0.89 ± 0.01. These results suggest that MST-Net achieved stable performance in the T2/T3 staging task for rectal cancer.

### Ablation experiment

To further evaluate the contribution of each component to T2/T3 staging of rectal cancer, experiments were conducted using Dataset A. The baseline model used the same backbone and classifier as MST-Net but removed both the MSFF module and the STAM. As shown in Table 3, the addition of either STAM or MSFF improved the AUC of the baseline model from 0.92 to 0.94, with statistically significant differences compared with the baseline model (*p* = 0.026 and *p* = 0.034, respectively). The full MST-Net model achieved the best performance, with an accuracy of 90% and an AUC of 0.95. Compared with the baseline model, MST-Net demonstrated a significant improvement in AUC (*p* = 0.003), suggesting that the integration of multi-scale feature fusion and spatial attention effectively enhanced model performance.

**Table 3:** Results of the ablation experiment

	Model	Acc (%)	Pre (%)	Rec (%)	Spe (%)	AUC	*p*-value*
Test set	Baseline	86	88	89	81	0.92	
	Baseline_STAM	88	90	91	83	0.94	0.026
	Baseline_MSFF	88	89	90	83	0.94	0.034
	MST-Net	90	92	91	87	0.95	0.003

*Acc* accuracy, *Pre* precision, *Rec* recall, *Spe* specificity, *AUC* area under the receiver operating characteristic curve

* *p*-value for comparison of performance (AUC) against baseline, calculated using the DeLong test

### Comparison with existing methods

**Fig. 4:**
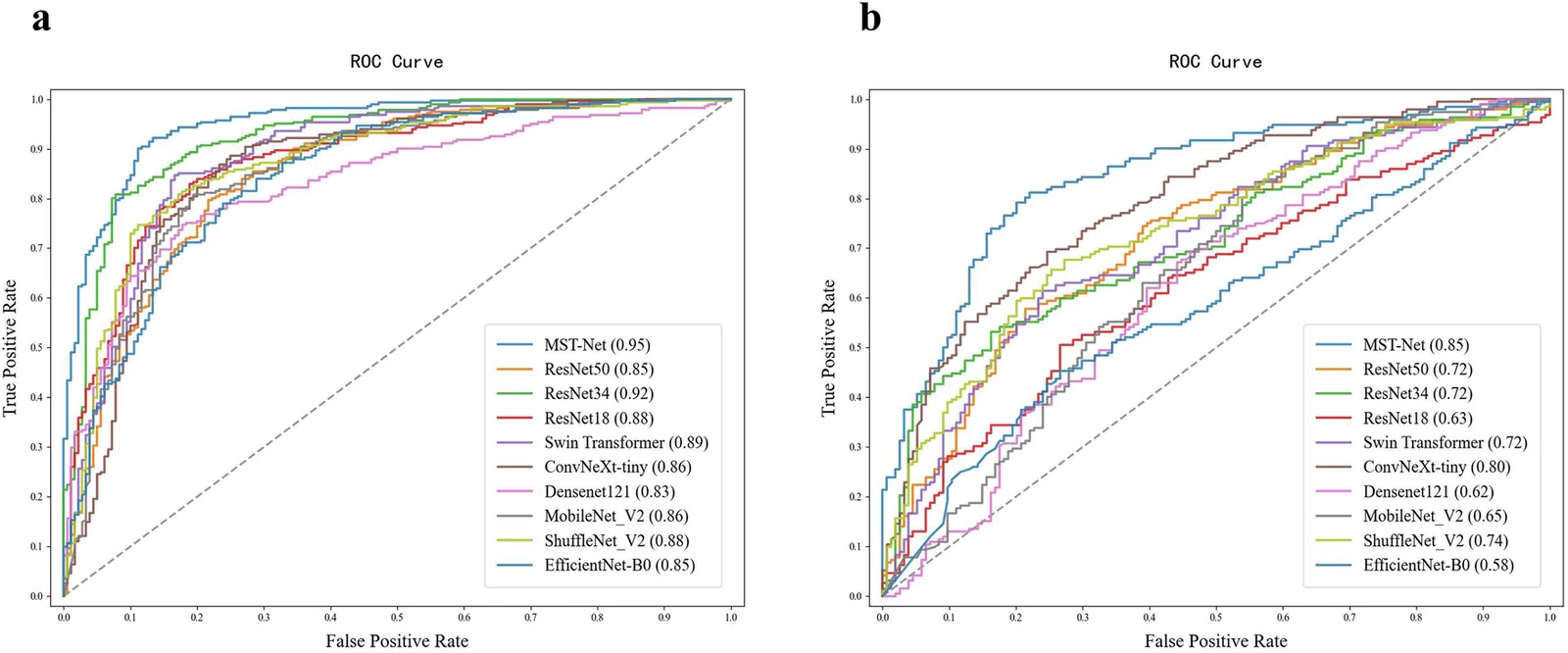
ROC curve comparison diagram. **a** The ROC curve on the test set; **b** the ROC curve on the external validation set

To evaluate the superiority of MST-Net, comparative experiments were performed against mainstream classification networks using the same internal test set and independent external validation set [23–29]. The quantitative results are summarized in Table 4, and the ROC curves are shown in Fig. 4. Differences in AUCs between MST-Net and each competing model were assessed using the DeLong test.

**Table 4:** Comparison with existing methods

	Model	Acc (%)	Pre (%)	Rec (%)	Spe (%)	F1 score	AUC	*p*-value*
Test set	ResNet18 [23]	81	88	79	83	0.83	0.88	< 0.001
	ResNet34 [23]	86	88	89	81	0.88	0.92	0.037
	ResNet50 [23]	80	80	89	65	0.84	0.85	< 0.001
	MobileNet_V2 [24]	80	86	80	81	0.83	0.86	< 0.001
	Densenet121 [25]	75	75	87	55	0.81	0.83	< 0.001
	EfficientNet-B0 [26]	79	81	87	67	0.84	0.85	< 0.001
	ConvNeXt-tiny [27]	83	83	91	72	0.87	0.86	< 0.001
	Swin Transformer [28]	84	89	85	83	0.87	0.89	< 0.001
	ShuffleNet_V2 [29]	81	88	79	84	0.83	0.88	< 0.001
	MST-Net	90	92	91	87	0.91	0.95	
Independent external validation set	ResNet18 [23]	58	62	65	49	0.63	0.63	< 0.001
	ResNet34 [23]	63	67	66	58	0.66	0.72	< 0.001
	ResNet50 [23]	63	64	78	49	0.70	0.72	< 0.001
	MobileNet_V2 [24]	62	65	69	53	0.67	0.65	< 0.001
	Densenet121 [25]	62	65	71	51	0.67	0.62	< 0.001
	EfficientNet-B0 [26]	57	59	71	40	0.65	0.58	< 0.001
	ConvNeXt-tiny [27]	66	67	77	53	0.72	0.80	0.030
	Swin Transformer [28]	60	62	73	43	0.67	0.72	< 0.001
	ShuffleNet_V2 [29]	64	67	70	56	0.69	0.74	< 0.001
	MST-Net	79	82	80	78	0.81	0.85	

*Acc* accuracy, *Pre* precision, *Rec* recall, *Spe* specificity, *AUC* area under the receiver operating characteristic curve

* *p*-value for comparison of performance (AUC) against MST-Net, calculated using the DeLong test

On the internal test set, MST-Net achieved the best overall performance, with an accuracy of 90%, precision of 92%, recall of 91%, specificity of 87%, F1 score of 0.91, and AUC of 0.95. MST-Net showed significantly higher AUCs than all competing models, including ResNet34, which had the second-highest AUC on the internal test set (AUC: 0.95 vs. 0.92, *p* = 0.037). For the remaining models, the differences were more pronounced, with all *p* < 0.001. On the independent external validation set, MST-Net also achieved the highest performance, with an accuracy of 79%, precision of 82%, recall of 80%, specificity of 78%, F1 score of 0.81, and AUC of 0.85. Its AUC was significantly higher than that of all other models, with the smallest difference observed for ConvNeXt-tiny (AUC: 0.85 vs. 0.80, *p* = 0.030), while all other comparisons showed *p*-values < 0.001.

Overall, MST-Net demonstrated superior and statistically significant performance compared with existing classification networks, supporting its robustness and generalizability for automated T2/T3 staging of rectal cancer on preoperative T2-weighted MRI.

### Model explainability

**Fig. 5:**
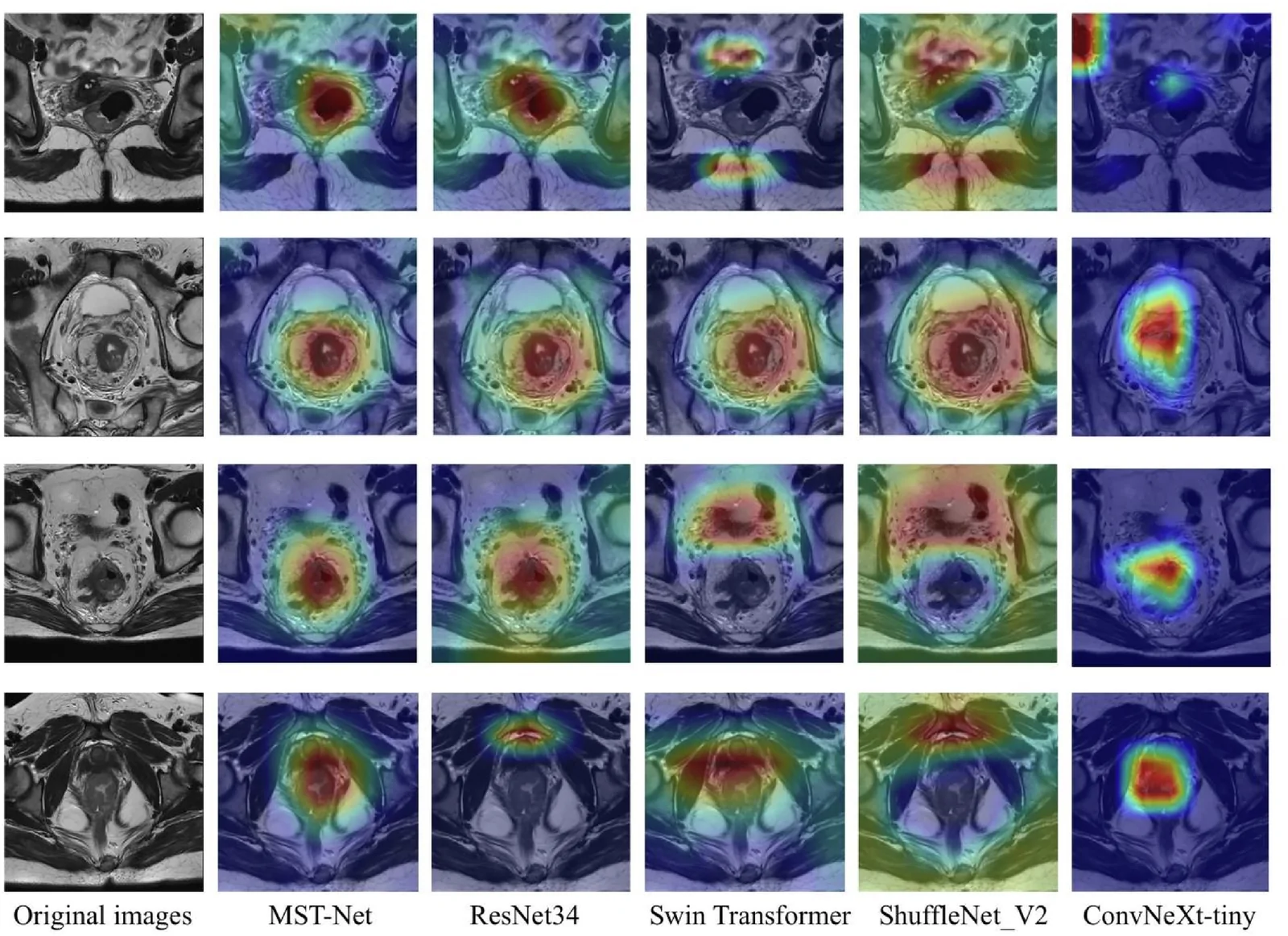
Heatmap comparison of different models

Grad-CAM++ was employed to provide visual interpretability for MST-Net [30]. As illustrated in Fig. 5, the activation maps visually highlighted tumor-related regions and surrounding anatomical areas relevant to T-staging. These findings provide qualitative support for the interpretability of MST-Net.

### Comparison of MST-Net, physicians, and physicians with MST-Net

To further evaluate the clinical applicability of MST-Net, we conducted a reader study involving three radiologists with different levels of experience: a junior radiologist, an intermediate radiologist, and a senior radiologist (labeled as Physician 1, 2, and 3). All radiologists independently assessed T2/T3 staging on the same internal test set and independent external validation set. During the unaided reading session, the radiologists were blinded to patients’ clinical information, pathological diagnoses, model predictions, and the assessments of the other readers. After a washout interval (3 weeks), the same radiologists reassessed the cases with the assistance of MST-Net. During the MST-Net-assisted session, the case order was randomized again to reduce recall bias. MST-Net assistance was provided in the form of model-predicted T stage and corresponding prediction probability. The diagnostic performance of radiologists before and after MST-Net assistance was compared using the McNemar test, and the results are summarized in Table 5 and the Supplementary Material (Table S4).

**Table 5:** Performance of physicians and MST-Net classification

	Method	Acc (%)	Pre (%)	Rec (%)	Spe (%)	F1 score	*p*-value*
Test set	Physician 1	69	77	71	67	0.74	< 0.001
	Physician 2	76	83	77	75	0.79	< 0.001
	Physician 3	85	88	88	81	0.88	0.051
	MST-Net	90	92	91	87	0.91	
Independent external validation set	Physician 1	65	70	65	65	0.67	< 0.001
	Physician 2	69	74	68	70	0.71	0.002
	Physician 3	72	78	70	75	0.74	0.044
	MST-Net	79	82	80	78	0.81	

*Acc* accuracy, *Pre* precision, *Rec* recall, *Spe* specificity

* *p*-value for comparison of performance (Acc) against MST-Net, calculated using the McNemar test

MST-Net demonstrated diagnostic performance comparable to that of senior radiologists and significantly outperformed junior and intermediate radiologists. Moreover, with the aid of the MST-Net model, diagnostic accuracy rates among junior and intermediate radiologists were significantly improved. These findings indicate that the model holds potential as a critical decision-support tool, enabling more comprehensive and precise diagnoses while reducing the risks of misdiagnosis and missed diagnosis. By providing highly consistent and objective evaluation results, MST-Net effectively alleviates the workload of clinicians and enhances diagnostic efficiency, which is particularly beneficial in resource-limited clinical settings. These findings highlight the potential value of MST-Net in improving objectivity and consistency in T2/T3 staging assessments of rectal cancer.

## Discussion

In this study, we developed and validated MST-Net, a segmentation-free automated deep learning model for differentiating T2 from T3 rectal cancer using preoperative T2-weighted MRI. The model demonstrated favorable performance in both the internal test set and the independent external validation cohort, suggesting its potential as an objective imaging-based tool for preoperative T-staging. By integrating pyramidal cross-stage feature fusion with a lightweight spatial attention mechanism, MST-Net was designed to capture both local tumor-wall details and broader perirectal contextual information, which are critical for distinguishing muscularis propria-confined disease from early extramural tumor extension.

Accurate preoperative staging of rectal cancer using MRI, particularly in distinguishing T2 from T3 disease, is fundamental for guiding appropriate therapeutic strategies [31, 32]. High-resolution MRI clearly delineates the depth of tumor invasion within the rectal wall and its relation to the mesorectal fascia and surrounding anatomy. T2-stage tumors are confined to the muscularis propria, while T3 tumors exhibit transmural extension into the perirectal fat [33]. This differentiation carries substantial clinical weight, as T2 lesions may be managed with local excision, whereas T3 tumors generally require neoadjuvant chemoradiotherapy before surgery to reduce the risk of involved circumferential resection margins. Staging inaccuracies can lead to either overtreatment or undertreatment, either of which may adversely impact patient prognosis. Thus, the clinical value of this study lies in its potential to mitigate management errors stemming from staging uncertainties, thereby supporting precision medicine in rectal cancer through more reliable imaging guidance.

Deep learning approaches using T2WI offer a promising avenue for automating the differentiation between T2 and T3 rectal cancer stages. Precise T-staging is essential to avoid unnecessary therapies in early-stage disease [34, 35]. In this context, MST-Net was developed to provide a segmentation-free automated framework for T2/T3 differentiation. Its pyramidal cross-stage feature fusion module integrates high-resolution shallow features with deeper semantic representations, while the lightweight spatial attention mechanism adaptively highlights staging-relevant regions and suppresses background interference. This design may improve sensitivity to subtle imaging features associated with early extramural invasion.

Compared with recent related work, the present study provides additional evidence supporting the value of deep learning for preoperative T2/T3 differentiation in rectal cancer. Lin et al developed a high-resolution T2WI-based DenseNet model in a multicenter cohort of 281 patients from four centers [25]; 255 patients from three centers were used for model development, and 26 patients from a fourth center were used for external testing. Their model achieved an AUC of 0.810 in the training cohort and 0.715 in the external test cohort, outperforming experienced radiologists in T2/T3 discrimination. In comparison, our study included 309 patients from two centers, with 270 patients from Center 1 used for model development and internal testing and 39 patients from Center 2 used for independent external validation. Although Lin et al included more participating centers, our study had a slightly larger total sample size and a larger external validation cohort. Methodologically, the two studies also differ in terms of workflow and clinical applicability. Lin et al used a DenseNet-based framework that required manual ROI delineation on selected image sections before preprocessing and model prediction. Therefore, their approach can be regarded as semi-automatic, and the manual segmentation step may increase the workload of radiologists and introduce operator-dependent variability. In contrast, MST-Net was designed as a segmentation-free automated image-based classification framework. After preoperative T2WI images are directly input into the network, the model can generate T-stage predictions without requiring manual ROI segmentation. This automated design may reduce operator dependence and better fit the routine clinical workflow. In addition, unlike conventional backbone-based classification models, MST-Net incorporates pyramidal cross-stage feature fusion and lightweight spatial attention, which may have contributed to the favorable performance observed in the present study. These differences suggest that the two studies provide complementary evidence for the feasibility of deep learning in T2/T3 discrimination, while the present study further emphasizes automation, workflow efficiency, and external validation.

From a clinical perspective, MST-Net may provide several practical benefits. First, it may reduce inter-reader variability, particularly in borderline T2/T3 cases where the distinction between desmoplastic reaction and true tumor extension is challenging. Second, the model may serve as a second-reader tool for radiologists by identifying cases with a higher probability of T3 disease, thereby supporting more consistent multidisciplinary treatment decisions. Third, because MST-Net uses conventional preoperative T2WI, it can be integrated into routine MRI workflows without requiring additional imaging sequences or contrast administration. This is particularly important for clinical translation, as models based on widely available sequences are more likely to be adopted across institutions. Furthermore, MST-Net demonstrated favorable clinical efficiency, with an average inference speed of 82.83 images per second, suggesting that it could provide rapid staging support in real-world clinical practice. Therefore, MST-Net may provide quantitative support for treatment stratification, especially in centers with variable levels of radiologist experience.

**Fig. 6:**
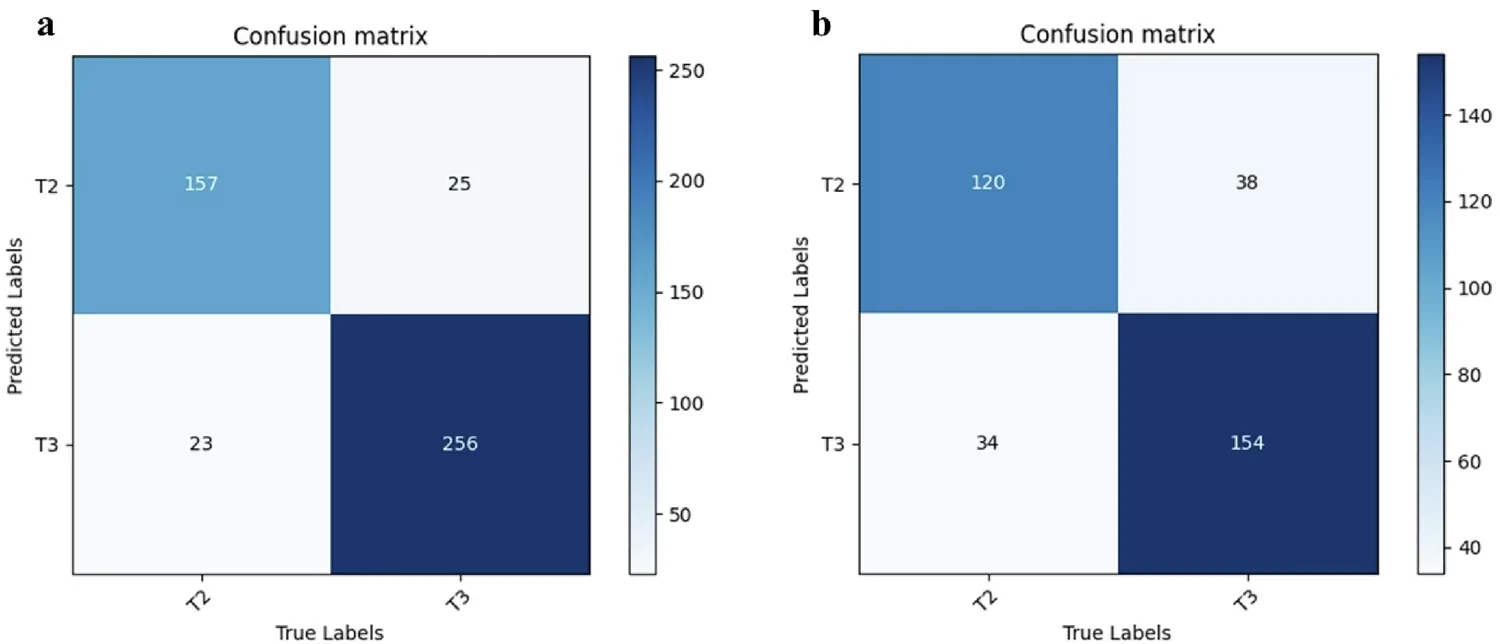
Confusion matrices for the proposed model. **a** The confusion matrix on the test set; **b** the confusion matrix on the independent external validation set

The confusion matrices further revealed clinically meaningful misclassification patterns. As shown in Fig. 6, analysis of these matrices reveals specific misclassification patterns. In the test set, 23 T2-stage images were misclassified as T3, while in the external validation set, 34 T2-stage images were misclassified as T3. A potential cause for this over-staging is the presence of peritumoral inflammatory reactions or proliferative fibrous tissue (desmoplasia), which can exhibit tumor-like signal abnormalities on T2WI (e.g., spiculated or strand-like signals), complicating the model’s ability to reliably distinguish these from true tumor invasion beyond the muscularis propria. In the test set, 25 T3-stage images were misclassified as T2. This under-staging may be associated with limited spatial resolution, small-volume extramural invasion, or tumor extension that is difficult to distinguish from adjacent normal tissue.

Although the automated staging model proposed in this study demonstrates satisfactory performance, several limitations warrant consideration. First, although an independent external validation cohort was included, the number of participating centers and the size of the external cohort remain limited. In addition, the internal dataset was acquired using United Imaging equipment; similarly, the external validation set was derived from another single center utilizing Siemens scanners. Differences in scanner vendors, acquisition parameters, signal-to-noise ratio, and spatial resolution may influence model performance. Larger multicenter studies with broader scanner and protocol heterogeneity are needed to further validate the robustness and generalizability of MST-Net. Second, this study relied on a single imaging modality. Although T2WI is the core sequence for local staging of rectal cancer and is widely available in routine clinical practice, T2WI alone may not fully capture tumor biological behavior. Future studies should explore multimodal fusion frameworks that incorporate diffusion-weighted imaging, dynamic contrast-enhanced MRI, clinical variables, serum biomarkers, and pathological features to provide a more comprehensive assessment of tumor invasion depth. Third, the present model was developed for binary T2/T3 discrimination and did not further subclassify T3 disease according to the depth of extramural invasion. Because the degree of extramural spread is also clinically relevant for risk stratification and treatment planning, future work should investigate whether MST-Net can be extended to more detailed T-subclassification tasks. Finally, prospective validation and reader-assistance studies are warranted to determine whether the model can improve radiologist performance, reporting consistency, and clinical decision-making in real-world practice.

In conclusion, MST-Net demonstrated promising performance for automated T2/T3 staging of rectal cancer using preoperative T2WI. By combining pyramidal cross-stage feature fusion with lightweight spatial attention, the model improved the extraction of staging-relevant spatial information and achieved stable performance in both internal and external validation. Compared with previous semi-automatic ROI-based deep learning approaches, MST-Net may offer a more efficient, reproducible, and clinically deployable solution for preoperative T2/T3 staging. These findings support its potential as an imaging-based decision-support tool for individualized treatment planning in rectal cancer.

## Conclusions

In summary, this study demonstrates that MST-Net enables automated, high-precision T-stage classification of rectal cancer from preoperative T2-weighted MRI. The model is specifically engineered to discriminate the critical T2/T3 staging boundary, a pivotal threshold for therapeutic decision-making. In comparative experiments, MST-Net surpassed existing mainstream staging methods, attaining consistent accuracy and recall rates exceeding 90% on the internal test set. Notably, the model maintained robust diagnostic performance on an independent external validation set, underscoring its strong generalizability and resistance to overfitting on narrow data distributions. These findings affirm that MST-Net provides not only a technically innovative solution for precise T2/T3 staging but also a potential decision-support tool for personalizing neoadjuvant therapy, guiding surgical resection strategies, and improving prognostic assessments. Consequently, the proposed framework exhibits considerable promise for clinical translation and practical application in oncologic imaging.

## Supplementary information

ELECTRONIC SUPPLEMENTARY MATERIAL

## Data Availability

The magnetic resonance imaging (MRI) data supporting the findings of this study are available from the corresponding author upon reasonable request.

## Abbreviations

ACC: Accuracy
AUC: Area under the receiver operating characteristic curve
CRC: Colorectal cancer
MSFF: Multi-scale feature fusion
Pre: Precision
Rec: Recall
Spe: Specificity
STAM: Spatial transformation attention mechanism

## References

[1] Bray F, Laversanne M, Sung H et al (2024) Global cancer statistics 2022: GLOBOCAN estimates of incidence and mortality worldwide for 36 cancers in 185 countries. CA Cancer J Clin 74:229–263 38572751 10.3322/caac.21834

[2] Li Q, Wu H, Cao M et al (2022) Colorectal cancer burden, trends and risk factors in China: a review and comparison with the United States. Chin J Cancer Res 34:483–495 36398126 10.21147/j.issn.1000-9604.2022.05.08 PMC9646460

[3] Han B, Zheng R, Zeng H et al (2024) Cancer incidence and mortality in China, 2022. J Natl Cancer Cent 4:47–53 39036382 10.1016/j.jncc.2024.01.006 PMC11256708

[4] Benson AB, Venook AP, Al-Hawary MM et al (2022) Rectal cancer, version 2.2022, NCCN clinical practice guidelines in oncology. J Natl Compr Canc Netw 20:1139–1167 36240850 10.6004/jnccn.2022.0051

[5] Gollub MJ, Blazic I, Felder S et al (2019) Value of adding dynamic contrast-enhanced MRI visual assessment to conventional MRI and clinical assessment in the diagnosis of complete tumour response to chemoradiotherapy for rectal cancer. Eur Radiol 29:1104–1113 30242504 10.1007/s00330-018-5719-1 PMC6358522

[6] Khasawneh H, Khatri G, Sheedy SP et al (2025) MRI for rectal cancer: updates and controversies—*AJR* Expert Panel Narrative Review. AJR Am J Roentgenol 224:e2431523 39320354 10.2214/AJR.24.31523 PMC12210418

[7] Nougaret S, Gormly K, Lambregts DMJ et al (2025) MRI of the rectum: a decade into DISTANCE, moving to DISTANCED. Radiology 314:e232838 39772798 10.1148/radiol.232838

[8] Jiang X, Zhao H, Saldanha OL et al (2023) An MRI deep learning model predicts outcome in rectal cancer. Radiology 307:e222223 37278629 10.1148/radiol.222223

[9] Yu L, Wang L, Tan Y et al (2019) Accuracy of magnetic resonance imaging in staging rectal cancer with multidisciplinary team: a single-center experience. J Cancer 10:6594–6598 31777588 10.7150/jca.32685 PMC6856893

[10] Lord AC, D’Souza N, Shaw A et al (2022) MRI-diagnosed tumor deposits and EMVI status have superior prognostic accuracy to current clinical TNM staging in rectal cancer. Ann Surg 276:334–344 32941279 10.1097/SLA.0000000000004499

[11] Horvat N, Rocha CCT, Oliveira BC et al (2019) MRI of rectal cancer: tumor staging, imaging techniques, and management. Radiographics 39:367–387 30768361 10.1148/rg.2019180114 PMC6438362

[12] Lin X, Zhao S, Jiang HJ et al (2021) A radiomics-based nomogram for preoperative T staging prediction of rectal cancer. Abdom Radiol 46:4525–4535 10.1007/s00261-021-03137-1 PMC8435521 34081158

[13] Lu HC, Wang F, Yin JD (2020) Texture analysis based on sagittal fat-suppression and transverse T2-weighted magnetic resonance imaging for determining local invasion of rectal cancer. Front Oncol 10:1476 33014786 10.3389/fonc.2020.01476 PMC7461892

[14] Patanè V, Atripaldi U, Sansone M et al (2025) MRI-based radiomics for preoperative T-staging of rectal cancer: a retrospective analysis. Int J Colorectal Dis 40:174 40778975 10.1007/s00384-025-04969-9 PMC12334464

[15] Wu QY, Liu SL, Sun P et al (2021) Establishment and clinical application value of an automatic diagnosis platform for rectal cancer T-staging based on a deep neural network. Chin Med J 134:821–828 33797468 10.1097/CM9.0000000000001401 PMC8104246

[16] Fan L, Wu H, Wu Y et al (2024) Preoperative prediction of rectal cancer staging combining MRI deep transfer learning, radiomics features, and clinical factors: accurate differentiation from stage T2 to T3. BMC Gastroenterol 24:247 39103772 10.1186/s12876-024-03316-6 PMC11299282

[17] Hou M, Zhou L, Sun J (2023) Deep-learning-based 3D super-resolution MRI radiomics model: superior predictive performance in preoperative T-staging of rectal cancer. Eur Radiol 33:1–10 35726100 10.1007/s00330-022-08952-8 PMC9755091

[18] Deng B, Wang Q, Liu Y et al (2024) A nomogram based on MRI radiomics features of mesorectal fat for diagnosing T2- and T3-stage rectal cancer. Abdom Radiol 49:1850–1860 10.1007/s00261-023-04164-w 38349392

[19] Ma X, Shen F, Jia Y et al (2019) MRI-based radiomics of rectal cancer: preoperative assessment of the pathological features. BMC Med Imaging 19:86 31747902 10.1186/s12880-019-0392-7 PMC6864926

[20] Zhang X, Zhang B, Wang B et al (2022) Automatic prediction of T2/T3 staging of rectal cancer based on radiomics and machine learning. Big Data Res 30:100346

[21] Tian C, Ma X, Lu H et al (2023) Deep learning models for preoperative T-stage assessment in rectal cancer using MRI: exploring the impact of rectal filling. Front Med 10:1326324 10.3389/fmed.2023.1326324 PMC10722089 38105894

[22] Wei Y, Wang H, Chen Z et al (2024) Deep learning-based multiparametric MRI model for preoperative T-stage in rectal cancer. J Magn Reson Imaging 59:1083–1092 37367938 10.1002/jmri.28856

[23] He K, Zhang X, Ren S et al (2016) Deep residual learning for image recognition. In: Proceedings of the IEEE conference on computer vision and pattern recognition (CVPR). IEEE, Las Vegas, pp 770–778

[24] Sandler M, Howard A, Zhu M et al (2018) MobileNetV2: inverted residuals and linear bottlenecks. In: Proceedings of the IEEE conference on computer vision and pattern recognition (CVPR). IEEE, Salt Lake City, pp 4510–4520

[25] Lin Y, Liu Y, Zhang X et al (2025) A high-resolution T2WI-based deep learning model for preoperative discrimination between T2 and T3 rectal cancer: a multicenter study. Acad Radiol 32:4524–4531 40221285 10.1016/j.acra.2025.03.048

[26] Tan M, Le QV (2019) EfficientNet: rethinking model scaling for convolutional neural networks. In: Proceedings of the 36th international conference on machine learning (ICML). PMLR, Long Beach, pp 6105–6114

[27] Liu Z, Mao H, Wu CY et al (2022) A ConvNet for the 2020s. In: Proceedings of the IEEE conference on computer vision and pattern recognition (CVPR). IEEE, New Orleans, pp 11966–11976

[28] Liu Z, Lin Y, Cao Y et al (2021) Swin transformer: hierarchical vision transformer using shifted windows. In: Proceedings of the IEEE/CVF international conference on computer vision (ICCV). IEEE, Montreal, pp 10012–10022

[29] Ma N, Zhang X, Zheng HT et al (2018) ShuffleNet V2: practical guidelines for efficient CNN architecture design. In: Proceedings of the European conference on computer vision (ECCV). Springer, Munich, pp 122–138

[30] Chattopadhay A, Sarkar A, Howlader P et al (2018) Grad-CAM++: improved visual explanations for deep convolutional networks. In: Proceedings of the IEEE winter conference on applications of computer vision (WACV). IEEE, Lake Tahoe, pp 839–847

[31] Arndt K, Vigna C, Kaul S et al (2023) Magnetic resonance imaging accuracy in staging early and locally advanced rectal cancer. Surg Oncol 50:101987 37717374 10.1016/j.suronc.2023.101987

[32] Milanzi E, Pelly RM, Hayes IP et al (2024) Accuracy of baseline magnetic resonance imaging for staging rectal cancer patients proceeding directly to surgery. J Surg Oncol 130:1674–1682 39233560 10.1002/jso.27852 PMC11849709

[33] El Khababi N, Beets-Tan RG, Curvo-Semedo L et al (2023) Pearls and pitfalls of structured staging and reporting of rectal cancer on MRI: an international multireader study. Br J Radiol 96:20230091 38696592 10.1259/bjr.20230091 PMC10546463

[34] Lu QY, Guan Z, Zhang XY et al (2022) Contrast-enhanced MRI for T restaging of locally advanced rectal cancer following neoadjuvant chemotherapy and radiation therapy. Radiology 305:364–372 35852424 10.1148/radiol.212905

[35] Fritz S, Killguss H, Schaudt A et al (2021) Preoperative versus pathological staging of rectal cancer—challenging the indication of neoadjuvant chemoradiotherapy. Int J Colorectal Dis 36:191–194 32955607 10.1007/s00384-020-03751-3

