# Supplementary material for: MST-Net: a multi-scale spatial transformation network for automated T2/T3 staging of rectal cancer on preoperative T2-weighted MRI
Source: Insights Imaging. 2026 Sep 4;17:220. doi: 10.1186/s13244-026-02377-3 (PMC13545167; doi:10.1186/s13244-026-02377-3)
Supplement: Supplementary file 1 [file 13244_2026_2377_MOESM1_ESM.pdf]

MST-Net: a multi-scale spatial transformation network for automated T2/T3 staging of rectal cancer on preoperative T2-weighted MRI

ELECTRONIC SUPPLEMENTARY MATERIAL

Appendix E1. Detailed structure of the Multi-scale Feature Fusion

Appendix E2. Detailed structure of the Spatial Transformation Attention Mechanism

Appendix E3. Evaluation Metrics

Fig. S1 Detailed structure of the Multi-scale Feature Fusion

Fig. S2 Detailed structure of the Spatial Transformation Attention Mechanism

Table. S1 Details of the Rectal Cancer Patient Dataset Division

Table. S2 MST-Net parameter details

Table. S3 Classification performance of MST-Net based on five-fold cross-validation results on the Dataset A

Table. S4 Performance of Physicians and Physicians+MST-Net classification in Test and Independent External Validation Set

Appendix E1

Detailed structure of the Multi-scale Feature Fusion

In traditional deep convolutional neural networks, feature propagation across different stages typically occurs independently. Features from shallow layers, which are characterized by higher spatial resolution, retain rich spatial details such as edges, textures, and local patterns. These features are well-suited for capturing local information and fine-grained characteristics. However, shallow features, while rich in spatial information, often lack sufficient contextual understanding [23]. In contrast, features from deeper layers, which exhibit lower resolution, encode stronger semantic information, such as object categories and global structures. These features are more suitable for high-level interpretation, although they sacrifice detailed lesion-related features.

To leverage the complementary strengths of multi-scale features, this study introduces a fusion strategy that integrates representations across different network stages. This approach enables the simultaneous capture of local anatomical details and broader contextual information, yielding a more discriminative feature set for staging. As outlined in the Appendix Fig. S1, the process first unifies the spatial resolution of all feature maps to 7×7 using adaptive average pooling. For shallow, high-resolution features (e.g., Stage 1: 56×56×64), pooling reduces spatial dimensions, after which a 1×1 convolution increases channels from 64 to 128, preserving fine details while expanding representational capacity. In contrast, for deep features (e.g., Stage 4:

512×7×7), a 1×1 convolution directly reduces channels to 128, ensuring that high-dimensional semantics do not dominate the fused output, a balance formalized in Equation (1)

Subsequently, the adjusted feature maps from each stage—now uniformly sized at 128×7×7—are concatenated along the channel dimension, yielding a fused 512-channel representation, as formalized in Equation (2). This design achieves complementary optimization: fine-grained local details (e.g., texture) from shallow layers are enriched by high-level semantic context (e.g., structural category) from deeper layers. This is facilitated through a channel-balanced architecture, where each stage contributes 25% of the output channels, coupled with resolution alignment. Consequently, the approach mitigates the common pitfall in conventional fusion methods where features from a single layer dominate the aggregated output. It is noteworthy that channel adjustment is implemented differently across stages: the first three stages undergo adaptive pooling followed by a 1×1 convolution, whereas the fourth stage utilizes only a 1×1 convolution.

$$F_i' = \begin{cases} Conv_{1\times1}\big(AdaptiveAvgPool2d(F_i, 7)\big), & i = 1,2,3 \\ Conv_{1\times1}(F_i), & i = 4 \end{cases} \tag{1}$$

$$F_{fused} = Concat(F_1', F_2', F_3', F_4') \in \mathbb{R}^{512\times7\times7} \tag{2}$$

Where F_i denotes the output features of the i-th stage of MST-Net, $F_i \in \mathbb{R}^{C_i\times H_i\times W_i}$, $i \in \{1,2,3,4\}$, Where C_1=64, H_1=56, W_1=56, C_2=128, H_2=28, W_2=28,

C_3=64, H_3=14, W_3=14, C_4=512, H_4=7, W_4=7. AdaptiveAvgPool2d(F_i,7) unifies the spatial dimensions to 7×7.

Appendix E2

Detailed structure of the Spatial Transformation Attention Mechanism

Following multi-scale fusion, the resulting 512×7×7 feature maps are processed by an STAM to accentuate diagnostically relevant regions while minimising background interference. This lightweight module dynamically generates a spatial weight map through a hierarchical convolutional structure and non-linear transformations. As depicted in the Appendix Fig. S2, the process begins with a 3×3 convolutional layer for local feature extraction and dimensionality reduction, followed by batch normalization and a ReLU activation. A subsequent 1×1 convolution then compresses the channel dimension to one, with a Sigmoid function normalising the output to a [0,1] range, as formalised in Equations (3) and (4). The final step involves an element-wise multiplication between this attention map and the original features, achieving adaptive spatial calibration (Equation (5)). By focusing dynamically on critical tumor sub-regions, STAM enhances feature discriminability more effectively than channel-wise mechanisms like the Squeeze-and-Excitation module, but with considerably lower parametric overhead.

$$Z_1 = ReLU\big(BN(W_1 * X + b_1)\big) \tag{3}$$

$$A = \sigma(W_2 * Z_1 + b_2) \tag{4}$$

$$Y = X \odot A \tag{5}$$

Here, BN (·) denotes batch normalization, while $*$ represents the convolution operation, $W_1 \in \mathbb{R}^{(C/4)\times C\times 3\times 3}$, $X \in \mathbb{R}^{C\times H\times W}$, $b_1 \in \mathbb{R}^{C/4}$. C, H, and W denote the number of channels, height, and width, respectively. W_1 and b_1 are the weights and bias of the first convolutional layer, where A denotes the spatial attention weight matrix. $W_2 \in \mathbb{R}^{1\times(C/4)\times 1\times 1}$, $b_2 \in \mathbb{R}$, σ denotes the Sigmoid function. W_2 and b_2 are the weights and bias of the second convolutional layer. $\odot$ denotes element-wise multiplication.

Appendix E3

Evaluation Metrics

$$\mathrm{Acc} = \frac{\mathrm{TP+TN}}{\mathrm{TP+FP+TN+FN}} \quad (6)$$

$$\mathrm{Pre} = \frac{\mathrm{TP}}{\mathrm{TP+FP}} \quad (7)$$

$$\mathrm{Rec} = \frac{\mathrm{TP}}{\mathrm{TP+FN}} \quad (8)$$

$$\mathrm{Spe} = \frac{\mathrm{TN}}{\mathrm{TN+FP}} \quad (9)$$

$$\mathrm{F1\ Score} = 2 * \frac{\mathrm{Pre*Rec}}{\mathrm{Pre+Rec}} \quad (10)$$

Among these, TP denotes positive samples predicted as positive by the model, FP denotes negative samples predicted as positive by the model, TN denotes negative samples predicted as negative by the model, and FN denotes positive samples predicted as negative by the model.

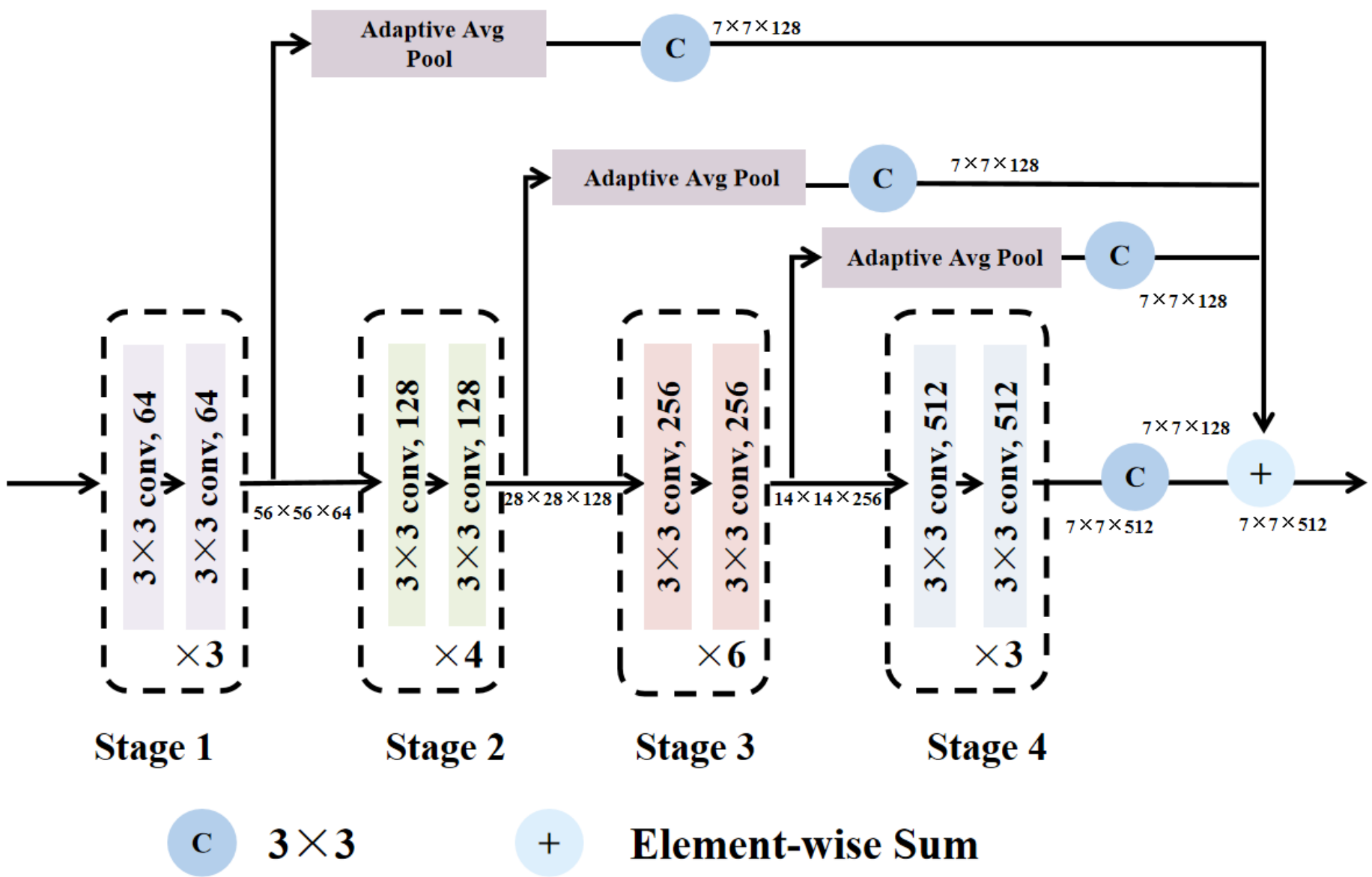

Fig. S1 Detailed structure of the Multi-scale Feature Fusion

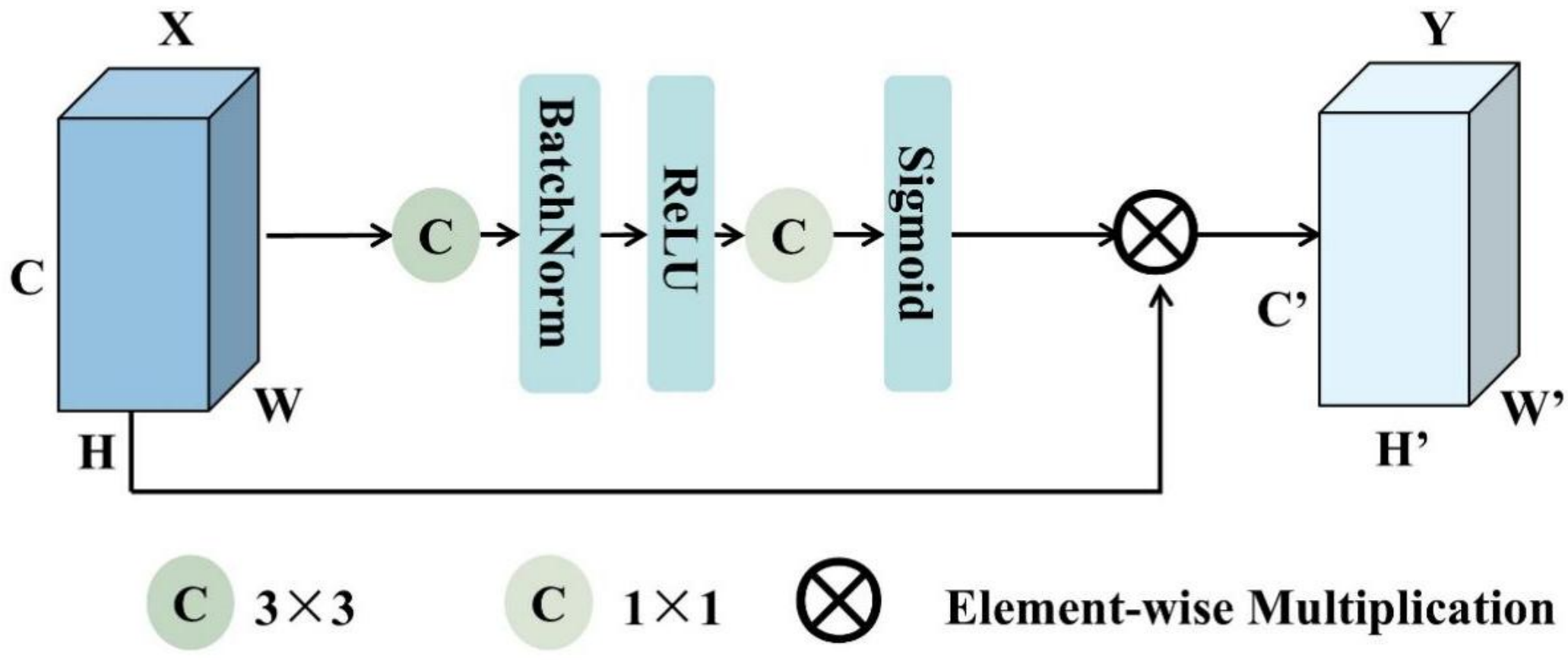

Fig. S2 Detailed structure of the Spatial Transformation Attention Mechanism

Table. S1 Details of the Rectal Cancer Patient Dataset Division

Stage Classification	Total Number of Patients	Dataset A		Dataset B
		Training set	Test set	External Validation set
T2	126 cases (1234 images)	88 cases (900 images)	22 cases (180 images)	16 cases (154 images)
T3	183 cases (1598 images)	128 cases (1125 images)	32 cases (281 images)	23 cases (192 images)
T2+T3	309 cases (2832 images)	216 cases (2025 images)	54 cases (461 images)	39 cases (346 images)

Table. S2 MST-Net parameter details

Parameter	Settings
Input dimensions	224×224
Optimizer	Adam
Learning rate	1e-3
Training epochs	200
Batch size	16
Number of subprocesses	4
Classification categories	2
Loss function	Cross-Entropy Loss
Total parameters	24.87M

Table. S3 Classification performance of MST-Net based on five-fold cross-validation results on the Dataset A

Model	Acc(%)	Pre(%)	Rec(%)	Spe(%)	F1	AUC
Fold 1	90	92	91	87	0.91	0.95
Fold 2	86	89	88	83	0.89	0.90
Fold 3	84	89	85	83	0.87	0.89
Fold 4	86	89	87	84	0.88	0.91
Fold 5	88	91	90	86	0.90	0.92
means ± SDs	86.8 ± 2.0	90.0 ± 1.3	88.2 ± 2.1	84.6 ± 1.6	0.89 ± 0.01	0.91 ± 0.02

Table. S4 Performance of Physicians and Physicians+MST-Net classification in Test and Independent External Validation Set

	Method	Acc(%)	Pre(%)	Rec(%)	Spe(%)	F1 Score	P value*
Test Set	Physician1	69	77	71	67	0.74	0.010
	Physician 1+MST-Net	77	83	78	75	0.81	
	Physician 2	76	83	77	75	0.79	0.002
	Physician 2+MST-Net	84	87	87	79	0.87	
	Physician 3	85	88	88	81	0.88	0.089
	Physician 3+MST-Net	89	92	90	88	0.91	
Independent External Validation Set	Physician 1	65	70	65	65	0.67	0.005
	Physician 1+MST-Net	75	79	75	75	0.77	
	Physician 2	69	74	68	70	0.71	0.008
	Physician 2+MST-Net	78	83	77	80	0.79	
	Physician 3	72	78	70	75	0.74	0.118
	Physician 3+MST-Net	78	82	77	79	0.80	

Note.— * P-value for comparing the performance (Acc) of the Physician and Physicians +MST-Net, calculated using the McNemar test.
